# The myogenic and neurogenic components of the rhythmic segmentation motor patterns of the intestine

**DOI:** 10.3389/fnins.2014.00078

**Published:** 2014-04-10

**Authors:** Jan D. Huizinga, Ji-Hong Chen

**Affiliations:** ^1^Department of Medicine, Farncombe Family Digestive Health Research Institute, McMaster UniversityHamilton, ON, Canada; ^2^Key Laboratory of Hubei Province for Digestive System Diseases, Department of Gastroenterology and Hepatology, Renmin Hospital of Wuhan University, Wuhan University Institute of Digestive and Liver DiseasesWuhan, China

**Keywords:** interstitial cells of Cajal, segmentation, motility disorders, motility, enteric nervous system, pacemaking

Almost all motor patterns in gut organs have as primary function the mixing of content. Although classical peristalsis is equated with propulsion, and this is certainly the case in the esophagus, the predominant effect in all other organs is mixing and exposing the content optimally to the mucosal surface, because the propulsion ends somewhere and the content is moving back; only very rarely does propulsion end with evacuation of content from the body. The segmentation motor pattern is different from peristalsis in that it contains only stationary or very short distance propagating contractions and hence is considered a specialized motor pattern for mixing and absorption. The segmentation motor pattern was described and illustrated by Cannon in 1902 based on X-ray observations and shown to be extremely rhythmic (Cannon, 1902). Alvarez was the first to find that the frequency of rhythmic segmenting contractions occurred at the frequency of a myogenic pacemaker and that in various regions of the intestine the frequency decreased in the same way as the pacemaker frequency decreased (Alvarez, 1914), suggesting a firm relationship between slow waves and segmentation. In 1968, Code and co-workers also recognized the role of slow waves in segmentation (Code et al., 1968); the slow waves were thought to go in and out of excitable regions of smooth muscle fibers. Ehrlein noted in 1987 that there were no electrical or mechanical features known that could distinguish peristaltic and segmental motor patterns (Ehrlein et al., 1987). In 2006, two major reviews on control of motility only very briefly mentioned segmentation: “The most basic small intestinal contractile pattern, segmentation, results from reciprocal inhibition and dis-inhibition of adjacent circular muscle” (Hasler, 2006) and “Oscillation of membrane potential through the slow wave cycle results in periods of high and low open probability for Ca^2+^ channels, and this naturally organizes the contractile pattern into a series of phasic contractions contributing to motility patterns such as peristalsis and segmentation” (Sanders et al., 2006). Recently, it was shown that spontaneous segmentation or decanoic acid-induced segmentation is associated with a waxing and waning of the slow wave activity that can occur prominently in the presence of nerve conduction block (Huizinga et al., 2014). Evidence was provided that waxing and waning developed when low frequency rhythmic transient depolarizations originating from interstitial cells of Cajal (ICC) associated with the deep muscular plexus (ICC-DMP) interacted with the omnipresent slow wave activity originating from ICC associated with the myenteric plexus (ICC-MP) through phase–amplitude coupling. That is, the phase of the low frequency activity modulated the amplitude of the higher frequency slow wave activity. Hence interacting myogenic electrical activities were seen to underlie the segmentation motor *pattern*. The segmentation motor pattern appeared to be associated with the *induction* of a low frequency component, causing minute rhythm clusters of contractions and a waxing and waning of the amplitudes of the individual contractions within a cluster (Huizinga et al., 2014). These clusters occurred prominently after decanoic acid in rats (Huizinga et al., 2014) or oleic acid in dogs (Ehrlein et al., 1987). In human small intestine studies, the post-prandial motility pattern can be quite variable but regular “cluster contractions” are often reported and seen to be mainly stationary (Hellstrom, 1995). In a study by Husebye (1999), the clusters were shown to have a minute rhythm, the contraction amplitudes within the clusters had a waxing and waning appearance with a frequency of ~10/min, hence occurring at the slow wave frequency (Husebye, 1999; Gallego et al., 2014). When a nutrient solution was given to healthy volunteers with or without 40 g/l ethanol, it was ethanol in particular that induced a low frequency component, the clustered contractions; the clusters occurred at 1 per 2–3 min and the individual contractions within the clusters at ~12/min with waxing and waning amplitudes (Schmidt et al., 1997). Hence also in the human small intestine, the slow wave frequency, as well as an additional lower frequency component, are reported in the post-prandial intestinal motor activity.

Although the segmentation rhythmicity has been associated with slow wave activity in many studies, other reports, primarily conducted using the guinea pig small intestine, explain segmentation solely on the basis of enteric neuronal activity. Important studies from Bornstein's laboratory provided evidence for the hypothesis that cholinergic motor neurons acting on muscarinic receptors periodically activate the musculature and that inhibitory neurons surround this contraction to finalize the motor pattern (Gwynne et al., 2004; Gwynne and Bornstein, 2007). Another study states that CCK and 5-HT are critical mediators in the regulation of segmentation (Ellis et al., 2013). Segmentation has also been suggested to result from a reduced degree of synchrony of AH neuron activity and sustained inhibition of the after-hyperpolarization (Ferens et al., 2007).

Can the myogenic and neurogenic hypotheses be reconciled? It is possible that control systems in the guinea pig are unique. However, there are arguments to be brought forward to suggest that slow waves may play a role in some if not all segmentation motor patterns of the guinea pig: The segmentation motor pattern is extremely rhythmic, 15–18 cycles per min according to figures shown in Gwynne and Bornstein (2007) which is within the range of the slow wave frequency of the guinea pig small intestine which is 11–19 cycles per min (Donnelly et al., 2001). The guinea pig small intestine is unique in that slow wave activity is not omnipresent but is *induced* by stimuli such as distention. Trendelenburg, in 1917, already recognized that the rhythmic peristaltic activity induced by distention in the guinea pig small intestine can proceed without a nervous conduction system (Trendelenburg, 2006). Slow waves in the guinea pig intestine are likely originating in networks of ICC, which are prominent and appear similar to other animal models (Zhou and Komuro, 1992; Burns et al., 1997; Lavin et al., 1998; Seki et al., 1998).

In the study of Gwynne and Bornstein (2007), segmental contractions were blocked by hyoscine (Gwynne and Bornstein, 2007). Does this prove that the segmentation is entirely neurogenic and that slow waves are not involved? No, because the expression of slow waves in tissues where slow waves have to be *induced*, the *inductor* often is the enteric nervous system. Hence, neural activity including muscarinic stimulation may induce ICC slow wave activity and provide excitation of the musculature at the same time. When muscarinic stimulation is involved in induction of slow wave activity, which then may be associated with segmentation, the activity will be hyoscine sensitive. In many organs in most species, omnipresent slow wave activity exists that will take part in the orchestration of motor patterns once the smooth muscle is stimulated above threshold for contractile activity. However, it has become clear in recent years that in addition to the omnipresent slow waves or, as is the case in the guinea pig intestine *instead* of omnipresent slow waves, slow wave activity occurs that depends on a stimulus. In the guinea pig, distention induces strong slow wave activity (Donnelly et al., 2001). In some guinea pig small intestine preparations, the distention-induced slow waves were abolished by atropine or TTX, in other preparations slow wave activity was not affected by these drugs, indicating that several distinct mechanisms can evoke the slow wave activity (Donnelly et al., 2001). In the rat and mouse colon, stimulus-dependent rhythmic transient depolarizations (similar to slow wave activity but called differently to avoid confusion and much lower in frequency) occur dependent on activation of L-type calcium channels and generated by a network of interstitial cells, the ICC-MP (Pluja et al., 2001). The rhythmic transient depolarizations occur in addition to the omnipresent slow wave activity, which is generated by the primary pacemaker cells of the colon, the interstitial cells of Cajal associated with the submuscular plexus (ICC-SMP). The ICC-MP activity takes part in the orchestration of colonic low-frequency propulsive contractions (Huizinga et al., 2011; Chen et al., 2013b). In the human esophagus, rhythmic contractile activity occurs when nitrergic inhibition is removed (Chen et al., 2013a). Vagal stimulation can initiate slow wave activity in the intramuscular ICC (ICC-IM) of the stomach (i.e., provide the pacemaker component) which together with activity from ICC-MP generate the full slow wave activity (Hirst et al., 2002).

Any hypothesis on the role of slow waves in contraction patterns has to recognize that slow waves themselves do not provide forceful contractions, forceful contractile activity usually depends on excitatory neural stimulation to generate general depolarization of the musculature to bring the slow waves that have propagated from ICC into the musculature above threshold for L-type calcium channel activation. Hence, neural activity is usually an active component of any segmentation motor activity in any organ of any species and therefore blockers of neural activity may inhibit segmentation motor patterns. This does not negate an essential role for slow wave activity.

The critical importance of the segmentation motor pattern makes it unlikely that only one mechanism exists to evoke it. Intraluminal nutrients in particular, in contrast to a non-caloric viscous meal, evoke segmental contractile activity in the dog intestine (Schemann and Ehrlein, 1986). Decanoic acid administered in this way in the guinea pig evokes a mixture of propulsion and segmentation (Ellis et al., 2013). Decanoic acid communicates with the epithelial layer and intraluminal decanoic acid-induced segmentation is inhibited by 5-HT_3_ and 5-HT_4_ antagonists and CCK antagonists. Most likely, 5-HT and CCK, released from enterochromaffin cells, stimulate enteric sensory neurons which then triggers excitatory activity in the ENS resulting in the motor patterns observed. The stimulatory action of motor neurons on the smooth muscle—ICC networks will then result in neurally driven activity that in part is orchestrated by ICC and smooth muscle intrinsic activities.

How activation of mucosal 5-HT receptors on enteric sensory neurons can lead to different motor patterns is not clear. It is possible that certain motor patterns depend on stimulation of serotonergic and purinergic receptors (Galligan, 2004). It is also possible that in conjunction with mucosal activation, actions occur through the blood stream on motor neurons, ICC and/or smooth muscle cells.

In summary, a number of studies have been published on the mechanisms underlying the segmentation motor pattern. Segmentation in all cases is observed as short-lived contractions that are propagating over a few mm, are often rhythmic in nature involving the whole intestine for a relatively long period or form clusters of segmental contractions for short periods. There is overwhelming evidence that the rhythmicity of segmentation is associated with ICC activity and recently, the classical segmentation motor pattern has been shown to occur in the presence of nerve conduction blockade. However, under most experimental conditions, and *in vivo*, the enteric nervous system provides an essential stimulus for the motor activity to develop and hence a variety of nerve conduction blockers or neural receptor blockers will inhibit segmentation activity. Whether or not a motor pattern occurs in response to nutrients or luminal distention is often determined by the response of the ENS to the stimulus, including sensory and motor neurons. This is also the case for segmentation, and several components of the ENS have been shown to be involved. This neural activity then works in concert with the ICC pacemaker activities to generate the motor pattern of segmentation.

## Conflict of interest statement

The authors declare that the research was conducted in the absence of any commercial or financial relationships that could be construed as a potential conflict of interest.
